# Understanding University Students’ Experiences, Perceptions, and Attitudes Toward Peers Displaying Mental Health–Related Problems on Social Networking Sites: Online Survey and Interview Study

**DOI:** 10.2196/23465

**Published:** 2021-10-05

**Authors:** Taewan Kim, Hwajung Hong

**Affiliations:** 1 Department of Industrial Design Korea Advanced Institute of Science and Technology Daejeon Republic of Korea

**Keywords:** mental health, social media, social support, peers, peer support, self-disclosure

## Abstract

**Background:**

College students’ mental health is at an all-time low. Students are increasingly disclosing their vulnerable, stigmatizing experiences on online social networking sites (SNSs). Peer support facilitated by SNSs can play a crucial role for the students in coping with mental health–related problems. Thus, it is imperative to understand how university students form perceptions, attitudes, and behaviors toward their peers who are dealing with mental health problems.

**Objective:**

This study aimed to provide a better understanding of how college students recognize, perceive, and react to signs of mental health problems in their peers on SNSs. Our ultimate goal in this study was to inform the design of SNSs that can facilitate online peer support.

**Methods:**

We conducted surveys with 226 students as well as semistructured interviews with 20 students at six universities in South Korea.

**Results:**

Of the 226 survey respondents, 150 (66.4%) reported that they recognized signs of a mental health problem on their friends’ SNS posts. However, a considerable number of respondents (62/150, 41.3%) were reluctant to offer support, even when they had identified friends who were at risk; this reluctance was due to a lack of knowledge or confidence and their desire to maintain a distance from at-risk peers to save their identity from stigmatization and to avoid emotional contagion online.

**Conclusions:**

Drawing on these results, we provide implications that could explain the construction of students’ perceptions regarding their peers’ mental health problems. We also provide design proposals for SNSs to serve as platforms that facilitate just-in-time and adaptive support exchanges among peers while mitigating stigma-related concerns.

## Introduction

### Background

College students’ mental health is at an all-time low because they are now exposed to a wide variety of stressors and pressures [1]. These students are also at a vulnerable life stage, which is frequently when the first episodes of serious mental health problems appear [2]. According to a 2018 World Health Organization survey of students in eight countries, 1 out of 3 students screened positive for a mental health illness and approximately 20%-35% of students might need treatment for mental health [3]. On the other hand, college is a place that provides students with an important opportunity to access a variety of mental health care resources, from clinical services to social support [4].

Social support and peer support—meaningful interactions that provide some sort of support, including emotional and instrumental support among fellow students who are of similar age and social status (ie, members of the same student population)—play a key role for coping with mental illness and maintaining well-being [5]. These peers may or may not have experienced similar mental health challenges. In this context, peer support includes a variety of interpersonal behaviors, including providing informational support (eg, providing advice and tips) and emotional support (eg, communicating care, confidence, and esteem) that can help fellow students with their mental health problems [6].

Social networking sites (SNSs) have served as a technology-mediated support system that facilitates the exchange of peer support [7,8]. Students are turning to SNSs to relieve their distress or to seek help from others by disclosing their vulnerable, stigmatizing experiences related to mental health problems [8]. A large body of research has addressed supportive online interactions with peers, which have been shown to help individuals who suffer from mental health problems attain improved mental well-being [9,10]. However, most of the research on peer support has focused on those who seek help and has not taken into account the potential benefits and risks of social support from the perspectives of those who are expected to provide support. Few empirical studies have examined how nonprofessional individuals—those who are susceptible to other people’s emotions and experiences—perceive online signals from at-risk friends. Thus, we need a better understanding of the experiences and challenges that these individuals face when identifying someone displaying signs of mental health problems on SNSs.

Furthermore, it is important to understand perceptions of the student population toward their friends experiencing mental health problems to tackle peer exclusion, lessen stigmatization, and further foster social support [11]. Public stigma [12] negatively affects those who could benefit from various mental health care resources, as they are reluctant to seek both personal and professional help [13]. Regarding the situation in which SNSs are used as a primary means of social interaction [14], we elucidate students’ perceptions toward their peers who they believe are experiencing mental health problems based on observing a variety of social interactions displayed on SNSs.

In summary, understanding peers’ viewpoints regarding mental health problems is crucial to empowering SNSs to serve as digital mental health support systems. The aim of this study is to investigate how college students perceive and react to their peers who display mental health–related challenges on SNSs and to identify factors that interfere with peer support. This paper documents the findings from online surveys and interviews conducted in six universities in South Korea. Note that we do not limit the term “mental health” to refer only to medically diagnosed illnesses. Rather, we consider all kinds of negative emotions and experiences, including those that make people feel vulnerable, including stress, loneliness, and minor depression.

### Related Work

#### Online Social and Peer Support for Mental Health

Individuals benefit from online social media as they interact [15], gain access to shared and tailored advice [10], and receive informational and/or emotional support from social network members [16]. Online social media can also provide useful resources for individuals with particular personality traits (eg, self-esteem issues [15]) as well as those who are affected by mental illness [17]. Individuals who experience mental health challenges often struggle to seek help because they feel embarrassed or have a fear of stigma and rejection [18]. Online social media platforms provide space for disclosing mental health issues with the benefits of convenience, privacy, and anonymity [19,20]; they also allow users to share difficulties and seek emotional support [21]. Social media helps college students, in general [22], and those who are suffering to attain improved mental well-being, in particular [9,10]. Thus, researchers have identified numerous factors that drive online social support [23] and have proposed computing platforms that would promote online mental health support from peers [17,24]. Despite the demonstrated importance of online social support, researchers have yet to empirically study the motives and challenges behind such support from the perspective of the peers who are expected to provide it.

#### Sensitive Disclosure and Responses on SNSs

SNSs allow users opportunities to address their mental health problems. Increasingly, college-aged people, who are the largest cohort of SNS users, have been posting sensitive disclosures on SNSs to relieve their psychological distress or to ask for help from others when they are in need [8]. A large body of research has addressed supportive online interactions with peers, which have been shown to improve the mental well-being of individuals who are suffering [9,10,21]. Earlier studies have addressed aspects of mental health–related SNS disclosures, including negative emotions [7], experiences of feeling stigmatized or vulnerable [25], depressive symptoms [26], curated self-harm images [27], the sharing of pro–eating disorder content [28], and even suicidal ideation [29]. Researchers have also revealed that sensitive disclosures garner supportive interactions between the discloser and the audience. For instance, due to their personal and intimate nature, sensitive disclosures (eg, personal narratives or references to an illness) have been shown to elicit more positive comments from peers on an SNS, as compared to less sensitive disclosures [25]. However, excessive negative expressions, such as statements about enduring loneliness, aversively affected the likelihood of both responses and likes [30]. Although a large number of previous studies have discovered the impact that sensitive disclosures have on general responses, few empirical studies have examined the holistic procedures and mechanisms through which the audience of a disclosure identifies, perceives, responds to, and even takes action for at-risk contacts on SNSs.

In summary, we found that a systematic investigation to determine how university students form perceptions, attitudes, and behaviors toward their fellow students’ mental health–related activities on SNSs was crucial in the study of human-computer interaction and health informatics. More specifically, we explored the following research questions:

- Research Question 1: Experiences—How do students discern their peers’ mental health problems through SNS content and activities? In other words, what kinds of SNS content and activities are considered by the viewers to be signals of mental health problems?
- Research Question 2: Attitudes—How do students react to the SNS activities of the peers who they believe are displaying mental health–related problems? What are the factors influencing these reactions?
- Research Question 3: Consequences—What are the consequences of viewing posts that are perceived as mental health–related issues on SNSs?

## Methods

### Overview

We conducted a two-phase study: Phase I involves a large-scale survey, and Phase II comprises semistructured interviews. Through an online survey of current students (N=226), we wanted to probe (1) the respondents’ experiences with friends who had signaled mental health issues on SNSs, (2) the perceived influence of such posts and activities, and (3) the types of support and responses the peers provided, along with their perceptions of the expected helpfulness of that support. We then conducted semistructured interviews with 20 of the participants to investigate why and how they decided to support or ignore their friends. We have limited our scope of analysis to semiprivate SNSs, such as Facebook, Instagram, and Twitter. We excluded private messenger services and public group forums.

### Recruitment

We targeted participants from various universities to overcome the limitations of the referral sampling method, in which students from various universities spread the word to individuals via SNSs and in which the gatekeepers of the universities’ online communities posted the study’s recruitment flyer. All undergraduate or graduate students were eligible to participate, provided that they were currently seeking a degree or would be matriculating into a degree program soon. We originally contacted 10 individuals at various domestic universities, and we eventually collected survey responses from 226 participants from six universities in South Korea. The recruitment posts featured links to an online survey and a consent form. We also added the participants to a raffle for a ₩5000 (US $5) gift card as compensation for their efforts. For the interview, we first sampled 102 participants from the group of survey participants who showed interest in participating in the interview session. Out of these 102 participants, 67 were initially selected based on the selection criteria. Of these, the final 20 interview participants were selected through random sampling. We selected interview participants with the following criteria: (1) participants who have detected mental health–related problems in others on SNSs, (2) participants who explicitly described details of the experience, and (3) participants who were determined that the researchers needed additional information to understand the experience in detail.

### Phase I: Online Survey

All survey participants were provided both demographic questions and semi–open-ended questions, the latter of which allowed them to select multiple responses and to enter text if necessary (Multimedia Appendix 1). In this way, the participants answered questions about (1) whether they had witnessed a person signaling mental health problems on SNSs, (2) why they were concerned about that person’s mental health, (3) what their relationship was to that person, and (4) what type of response they provided. We also asked the participants to answer several questions about their perceptions of closeness, responsibility, susceptibility, and helpfulness using a 5-point Likert scale. We iteratively developed these questionnaire items based on the existing literature; our intent was to examine the factors that influenced the participants’ degree of willingness to respond to mental health–related disclosures [31]. In turn, we determined the following four factors that could influence students’ perceptions and attitudes toward individuals who signaled mental health issues on SNSs:

1. Closeness [32]: “To what extent did you feel that you were close with the person you mentioned above?” Studies have revealed that the degree of relationship closeness between two individuals influences the extent to which intimate disclosures between them are positively received [23]. In the same vein, disclosing intimate information to a stranger can be seen as socially unacceptable behavior; such disclosures are often thought of as inappropriate [33]. As previous studies have shown that closeness affects the acceptance and recognition of such disclosures, we sought to investigate whether this factor also affects the willingness to support the discloser.
2. Responsibility [34]: “To what extent did you feel that you should respond to that person?” Because we focused on publicly shared content for which no recipient was specified, no particular person was obligated to respond [7]. We examined the extent to which the participants felt responsible for reacting to mental health–related disclosures.
3. Susceptibility [35]: “How did that person’s disclosures or other activities influence your emotions?” According to Forgas [35], a discloser reveals more intimate information when in a negative mood. However, little research has been done to examine how these intimate disclosures affect the viewers’ emotions or attitudes. Thus, we investigated how viewers’ emotional susceptibility affected their willingness to respond to disclosures.
4. Helpfulness [34]: “To what extent did you feel that your response or support would be helpful to that person?” We considered how confident the participants were in responding to the disclosure or in providing support for the discloser, as well as whether the perceived helpfulness of their actions would impact their willingness to respond.

### Phase II: Semistructured Interviews

We conducted follow-up interviews with 20 of the survey participants to elucidate the perceptions and conception of social support from the viewers’ perspectives. Of the 226 respondents in the Phase I survey, 20 participants were selected. Specifically, we wanted to elicit detailed and accurate narrative accounts of students’ experiences. These interviews lasted from 40 to 60 minutes. To help them recall past experiences, we asked the participants to browse their own SNS accounts. We asked them to tell us about some posts and activities that may have signaled that an individual was facing mentally challenging situations, such as Facebook messages or Instagram images that reflected unstable mental health conditions (eg, depressive states, suicidal ideations, or requests for help). We then asked the participants how they reacted to the posts, how that experience impacted their perceptions of the poster, and about their chosen methods of support. Our interview protocol and questionnaires were reviewed by three clinical psychologists working in a university’s health care center. Based on the interview guide (Multimedia Appendix 2), two researchers who conducted the interviews ran two rehearsal sessions. The interviews were conducted in a face-to-face format in a quiet lab where only an interviewer and an interviewee could talk privately.

### Analysis

We analyzed the survey questions, which contained single-selection, multiple-selection, and Likert-scale responses, using descriptive statistics and Mann-Whitney *U* tests. For quantitative analysis, we used Prism (version 8; GraphPad Software). We also examined the responses to the open-format survey questions and the interview transcripts using the qualitative data analysis software ATLAS.ti (version 7; Scientific Software Development GmbH) for inductive thematic analysis [36]. Interviews and surveys were conducted and transcribed in Korean, and analyses were also conducted in Korean. This process allowed us to analyze the original responses as accurately as possible. For the responses cited in the paper, an English-Korean bilingual-speaking researcher first translated interview responses into English and then revised them through consultation with a proofreading expert. Two researchers independently coded the responses and transcripts to iteratively formulate possible themes and then compared their codes. The coding process was recursive; it ended when the researchers considered the themes to have stabilized, and a set of distinct themes emerged.

### Ethical Considerations

We acknowledge that this study addresses several ethical concerns, even though the researchers’ institutional review board approved it in March 2017. In this study, we did not investigate the “posters,” those who produced the references to mental health issues, because our primary research focus was on understanding the perspective of the “viewers,” those who were concerned about the posters. Thus, we did not directly interact with highly vulnerable individuals who might have mental illness diagnoses. However, we acknowledge that mental health problems are very common among students. Thus, it is quite likely that some of the participants have also struggled with undiagnosed disorders (eg, depression), given the crisis regarding such disorders that has struck college campuses nationwide [37]. Note that during the interviews, 3 participants indicated that they had been diagnosed with major depressive disorder. To reduce the chances that our participants would identify the original posters, we asked the participants not to show or tell us any identifying information about the posters (eg, their names or SNS accounts).

## Results

### Survey Results

#### Overview

The survey participants comprised 226 students from six institutions of higher education in South Korea. The collection of survey answer data was conducted from March 27 to April 15, 2017. Table 1 shows the overall survey participant demographics. The participant sample included undergraduate students (n=178, 78.8%), master’s students (n=26, 11.5%), and doctoral students (n=22, 9.7%). Regarding gender, 91 (40.3%) participants were male, 133 (58.8%) were female, and 2 (0.9%) did not specify gender. Respondents were between 19 and 30 years old (mean 22.4, SD 2.6). The survey began with the question, “Have you ever seen a person whom you know displaying signs of a mental health problem on his/her SNS?” The majority of survey respondents (n=150, 66.4%) reported that they had seen such activities. Some participants (n=18, 8.0%) referenced friends who had been diagnosed with mental illnesses, including major depressive disorder (n=9, 50%), schizophrenia (n=1, 6%), eating disorder (n=1, 6%), posttraumatic stress disorder (n=1, 6%), anxiety disorder (n=2, 11%), and unspecified (n=4, 22%).

The interview participants comprised 20 students from among the survey participants: 11 (55%) female and 9 (45%) male. All 20 interviewees were born and raised in South Korea. Their knowledge and awareness of mental health problems varied. During the interview process, 3 (15%) participants voluntarily disclosed having a past diagnosis of mental illness. In addition, 1 (5%) participant noted that his former girlfriend had diagnosed him with depression, and another participant (n=1, 5%) had a family member who had been diagnosed with a mental illness. In total, 3 (15%) of the interviewees were graduate students—2 were in a master’s program and 1 was in a doctoral program—and the rest were undergraduate students. Interviews were conducted from April 12 to July 26, 2017.

**Table 1:** Demographic information about survey participants.

Demographic		Participants (N=226), n (%)
**Gender**		
	Male	91 (40.3)
	Female	133 (58.8)
	Not specified	2 (0.9)
**Age in years**		
	22-24	163 (72.1)
	25-27	46 (20.4)
	28-30	17 (7.5)
**Degree course**		
	Undergraduate	178 (78.8)
	Master’s student	26 (11.5)
	Doctoral student	22 (9.7)

#### Types of SNS Content and Activities Displaying Mental Health Issues

A total of 150 respondents out of 226 (66.4%) reported that they had seen a person displaying signs of a mental health problem on his or her SNS. These respondents found that the media the posters used were mostly text messages (n=132, 88.0%), with some images (n=7, 4.7%) and other types of content, such as shared links (n=11, 7.3%). These posts reflected each poster’s negative feelings, stigmatized experiences, or moods, and they made him or her appear to be suffering. For instance, Participant #77 perceived a friend’s Facebook post, which contained a photograph of a celebrity who had died by suicide, as implicit suicidal ideation. In addition, 18.7% (n=28) of the respondents identified signs of mental health problems by tracing a poster’s activities over time, such as a constant liking of content mentioning depression (Participant #59) or a sudden burst of Facebook updates (Participant #29). The detailed reasons for how and why these respondents became concerned about the posters’ mental health conditions are reported in the Qualitative Analysis section.

#### Types of Reactions

After taking a closer look at the types of reactions that the peers provided to the posters, we found that a considerable number of survey respondents who had seen mental health–related posts on SNSs (62/150, 41.3%) did not respond to the posts or provide any other support, even though some (22/150, 14.7%) still had concerns. Of the 88 respondents who reacted to the posts, 30% (n=26) expressed their emotions using simple communicative features of SNSs (eg, the “like” button options or crying emoticons), 18% (n=16) wrote short comments on the posters’ SNS feeds to express support (eg, “It will get better”), 16% (n=14) sent private messages to the posters, 7% (n=6) made appointments to meet with the posters, 2% (n=2) offered specific advice, and 1% (n=1) researched resources on behalf of the posters (eg, by calling a counselor).

#### Students’ Perceptions That Can Affect Support

As we reported above, a considerable number of survey respondents reported that they did not respond to the friends’ posts signaling mental health problems. Thus, we wanted to determine how these viewers’ perceptions differed from those who did react. The average scores for these perception properties were higher for peers who provided support than for those who did not. To determine whether there were significant differences between the two groups, we conducted a Mann-Whitney *U* test (two-tailed, reported with a *U* score and a *P* value). Table 2 summarizes the results for all assessments.

**Table 2:** Comparison of participants’ perceptions.

Perception	Participants who provided support (n=88)			Participants who did not provide support (n=62)		*U* test	*P* value
	Mean (SD)	Median (IQR)	Mean (SD)		Median (IQR)		
Perceived closeness	2.86 (1.22)	3 (2)	1.74 (0.81)		2 (1)	1327	<.001
Perceived responsibility	3.28 (1.01)	3 (1)	2.24 (0.93)		2 (2)	1316	<.001
Perceived susceptibility	3.09 (1.07)	3 (2)	2.96 (1.19)		3 (2)	2353	.14
Perceived helpfulness	3.09 (1.07)	3 (2)	2.67 (1.27)		2 (2)	1435	<.001

We found that peers were significantly more likely to support others if they felt more responsible (*U*=1316, *P*<.001), were closer to the poster (*U*=1327, *P*<.001), or believed that their support would be helpful (*U*=1435, *P*<.001). Susceptibility did not affect the likelihood of providing support (*U*=2353, *P*=.14). There was no significant difference between male and female participants. Overall, the viewers’ perceived responsibility, their closeness to the posters, and their perceptions of the expected results of their support all impacted the likelihood that they would provide social support. Keeping these high-level findings in mind, we move on to the interview data, through which we aim to understand the detailed contexts in which these patterns occurred.

### Qualitative Analysis

#### Overview

Our findings indicate that the participants based their perceptions of others’ mental health conditions on a variety of signals (Table 3). Many participants used the communicative features of SNSs to help their friends who appeared to be suffering from mental health issues. We also identified several challenges that led to most participants refraining from providing such support. We reported participant quotes using identity codes, the type of SNS they mentioned, and the source of the quote.

**Table 3:** Signals that raised participants’ concern about others’ mental health problems. Codes or categories are not mutually exclusive.

References to mental health concerns	Participants, n (%)		Detailed descriptions	Examples of quotes or cases reported
	Survey (n=150^a^)	Interview (n=20)		
Disclosed mood and diagnostic history	121 (80.7)	15 (75)	Negative mood, from depressive symptoms to suicidal feelings Use of swearing or abusive or offensive language Disclosure of mental illness diagnosis (eg, alcohol problems and anxiety disorder) and experiences or desire for treatment, such as counseling or medication	“He wrote he’s seeking information about a psychiatric service on campus because he was not getting along well at school.” (Participant #140) “My friend’s post, ‘I’m a loser. I feel I will never be successful,’ with no details frightened me.” (Participant #34)
Personal life struggles and stigmatized experience	53 (35.3)	9 (45)	Description of severe difficulties and challenges that negatively impact a poster’s life (ie, family disruption, romantic breakups, interpersonal difficulties, and personal concerns)	“He considered dropping out of college due to feeling overwhelmed.” (Participant #141) “After breaking up with his girlfriend, he expressed how he felt devastated every night.” (Participant #88)
Encoded content implying mental health problems	10 (6.7)	4 (20)	Implicit signals, such as the use of literary references or visual images implying mental health problems	“My ‘bro’ writes many songs. He often posts very dark and macabre lyrics. Sometimes, he posts a video streaming of himself singing that song. It seems weird.” (Participant #51) “It was a poem about death. She kept posting such things on her Instagram.” (Participant #69)
Gaps between offline and online identity	22 (14.7)	4 (20)	Gaps between online identity appearing to be at odds with his or her offline identity	“I felt that it was written by a totally different person. The tone in the post was completely different from what he used to be like.” (Participant #115) “It feels like a ‘drunken Facebooking.’ As far as I know, he’s always sober. He never drinks.” (Participant #63)
Social interaction and activity log	29 (19.3)	6 (30)	Constant usage of social media as a forum for expressing negative feelings A drastic change in social media activities (eg, a sudden burst of Facebook updates) and unusual communicative patterns	“He always shares such a depressive thing every day. He is ‘liking’ a tremendous number of things that are extremely negative.” (Participant #29) “He’s weird. He writes things that no one cares about. If there’s no reply, he then deletes his post. I have seen this [his repetitive posting and deleting activity] happening so many times.” (Participant #12)

^a^Respondents who reported that they had seen a person displaying signs of a mental health problem on social networking sites.

#### Experiences: How Do Students Discern Their Peers’ Mental Health Problems Through SNS Content and Activities?

Our participants cited a number of signals that led them to think that their friends might have mental health problems. These ranged from explicit references, such as mood disclosures in SNS content, to implicit references, such as inconsistent offline and online personality traits. Table 3 summarizes these signals, gives a detailed description of each one, and lists examples provided by both survey and interview participants.

The explicit disclosure of negative emotions provided a clear signal that led our participants to become concerned about a poster’s mental health. The disclosed moods included feeling exhausted and overwhelmed (37/170, 21.8%), depressed (35/170, 20.6%), lonely (19/170, 11.2%), angry (15/170, 8.8%), hopeless (9/170, 5.3%), and other (6/170, 3.5%). One interview participant responded that a poster’s symptoms appeared to be serious if they displayed negative feelings without a detailed explanation of such feelings:

She [a poster] updated her Facebook like, “I’m useless. There’s no reason for why I have to live”...When I saw this post, I felt she was at risk though she never gave a reason for saying this. It seemed like a very conclusive remark. That caused me to be really worried about her.

Participant #67, Facebook, interview

Some participants also considered the disclosure of negative experiences as a reliable source for confirming a poster’s unstable mental state. They shared examples of experiences that made the poster seem vulnerable, including disclosures of interpersonal strife (18/170, 10.6%), low academic performance (9/170, 5.3%), relationship breakups (3/170, 1.8%), family issues (2/170, 1.2%), financial hardship (2/170, 1.2%), and other challenges (19/170, 11.2%). Disclosures about negative moods, diagnostic history, and stigmatized experiences were often used to recognize the state of a poster’s mental health, but some participants cited more implicit signals, such as the use of literary references or visual images [25].

In our survey, most of the participants (129/150, 86.0%) reported having a personal relationship with the suffering individuals in real life (eg, a roommate). Thus, the participants had already constructed an identity for the poster through offline interactions. However, if they recognized that the poster’s online identity was at odds with his or her offline identity, they became concerned:

As far as I know, my friend is a very kind and positive person. He loves his friends. He has often told me that sincere friendships are the most important thing in his life. However, I just found a sudden update on his Facebook saying that his life was meaningless. I felt like he was a totally different person online.

Participant #84, Facebook, interview

A series of SNS data can provide a window for understanding an individual’s usage pattern and motives for usage [38]. Some participants pointed to an exceptionally high frequency of updates as one source of concern. This finding is consistent with the results of a prior study that revealed that a depressed user group showed an increased rate of wall posts [39]. In this regard, the participants did not rely on a single post but rather made an inference by observing a poster’s usage characteristics over a longer time period:

He always shares his negative feelings, but no one reacts to him: no likes, no comments. If there’s no response for a couple of days, he then deletes the original post. He has done this kind of thing many times as far as I can tell.

Participant #13, Facebook, interview

#### Attitudes: How Do Students React to the SNS Content and Activities of the Peers Displaying Problems? What Are the Factors Influencing These Reactions?

#### Overview

Our findings show that SNSs were used as a mediating tool to recognize others’ mental health problems in various ways. Meanwhile, our survey results reveal that a significant percentage of the peers remained inactive. In this section, we give our attention to identifying reasons for viewers’ reactions toward the SNS content and activities of the poster who displayed mental health problems through SNSs.

#### Attitudes Toward Social Support and Self-disclosure in SNSs

Participants reported negative beliefs about the concept of social support using SNSs. Furthermore, they mentioned that mental health issues would not be treated simply by leaving a short comment or pushing the “like” button. A total of 44.7% of the respondents (67/150) in the survey answered that it would be “very unhelpful” and “somewhat unhelpful.” Many participants expressed low expectations and even negative feelings regarding reacting to the poster disclosing sensitive matters on SNSs:

I am not sure these kinds of SNS reactions could make my sister better.

Participant #37, Facebook, survey

Participants had a negative perception of self-disclosure associated with stigma in SNSs considered as public places. Therefore, many of the participants were reluctant to react because their reactions would negatively impact the poster. They warned that their reactions might make the poster rely on online interactions rather than seek professional help. In some cases, participants thought that the poster just wanted to draw attention:

He keeps posting depressed posts to aggregate comments and likes. I think this cycle will make him more depressed.

Participant #11, Facebook, survey

#### Challenges in Responding to the Poster

Participants said that they did not want to directly interact with someone who might need mental health care, due to their own lack of expertise and confidence. One participant reported that he had no idea what to say and argued that even an indifferent response would be worse than no response:

I am still not sure whether I could help others...I don’t know anything about mental health. I can’t even say something like “hang in there,” because I’m afraid that my naïve response may hurt someone who needs serious care.

Participant #13, Facebook, interview

While participants could recognize posters’ signals of being at risk, it was not always easy for them to take action in response. They experienced feelings of guilt about watching another’s social feed reflecting private matters. Thus, one participant decided to remain silent:

Note that I am neither a stalker nor a lurker. But her unusual social media activities often catch my eye. Seeing what others are doing online is like reading another person’s diary. So, unless requested, I won’t respond. I will be quiet.

Participant #67, Facebook, interview

We found that many participants wanted to keep away from the person displaying mental health issues on SNSs. Participants mentioned that they do not add a comment or a “like” because they do not want to be part of the posts. By doing that, they intend to keep some distance from the poster. They also mentioned that the SNS environment where everyone can see their social interaction log (eg, comments, “likes,” and shares) also makes them more careful:

I think that others will also see me as a similar type of person if I write a comment on his postings...So, I would not respond to such kinds of weird postings.

Participant #63, Facebook, interview

Another way of keeping a distance from the poster was to unfollow the poster. One participant who recovered from depression a few years ago reported that she unfollowed her friends who constantly post depressive content. She did so because the posts reminded her of when she was depressed in the past. Another participant reported that he blocked one of his friends who “contaminated” his news feed with all kinds of negative matters. While the previous study showed that the main reason for keeping a distance was because of induced fear (ie, fear that the person will do something violent) [11], four findings show that people wanted to keep a social distance to save their identity from stigmatization and avoid emotional contagion. Thus, many of the participants decided to view their friends’ posts without directly interacting in any way, known as lurking, to maintain distance from the poster.

#### Consequences: What Are the Consequences of Viewing Posts That Reveal Mental Health Issues on SNSs?

#### Providing Social Support

While many participants did not respond for a variety of reasons, some have used SNS features to provide appropriate help. Participants used SNSs to track whether someone they care about might be suffering from a mental health problem and even to provide support in a timely manner. When participants detected a problem, they leveraged their background knowledge about the poster to respond. For instance, one participant, whose friend was struggling with an eating disorder, reported an example of how she could help her friend:

She looks okay on her Instagram, but I’m the only one who knows that she’s struggling with an eating disorder. So, whenever she posts food images, such as sushi or cakes, I really worry about her. Others would think, “Oh, it looks delicious, and she might want to share her happy moment with others.” But I cannot think that way. So, I call her immediately.

Participant #67, Instagram, interview

Some participants acknowledged that it was difficult to talk with a poster about his or her mental health issues even if they were close to the poster. We found that some participants contacted a poster’s family member or close friend if they noticed the poster was at risk. Two of the participants detected an implicit signal of suicide in their friends’ posts. One called the friend’s sister directly, and the other one sent a direct message to a member of the friend’s family. Some participants encouraged their friends to seek help and coping resources. Rather than trying to pressure their friends into treatment, they deliberately approached them with information:

She posted something that implied suicidal ideation, but I could not say, “You have a problem. You should go to the center.” Instead, I asked her, “Why don’t we go to the counseling center? I feel I am overwhelmed. I want to go there with you.”

Participant #94, Facebook, interview

This participant sought to help her friend in a way that would make her feel comfortable. In turn, she was thankful that her friend was able to be diagnosed with depression and began regularly visiting a psychiatrist.

#### Fatigue and Stigma From the Posts

In contrast to the discerning peers who carefully supported suffering individuals in a timely manner, a considerable number of participants did not proactively provide support, even when they noticed friends at risk due to various reasons. SNS curation features helped our participants identify their peers’ problems. However, constant displays of negative feelings and experiences resulted in audience fatigue. Such fatigue could result in a high threshold for concern, meaning that peers could become less troubled by those who keep expressing problems. Also, an individual’s disclosure of such negative aspects could lead to a negative stereotype or a fixed identity for the person as someone who is always depressed:

If you know someone who keeps disclosing negative feelings on Facebook, you’ll say, “Well, that is what he always does. A blue guy.” So, no matter how serious he is, [such repetitive behavior] will be likely to mute the signal.

Participant #72, Facebook, interview

## Discussion

### Overview

Our online survey and interview study provided a rich description of students’ attitudes toward, and identification experiences with, peers who display signals of mental health problems on SNSs. In this section, we discuss further implications of the findings by extending previous studies to the research community. We then present design considerations for social media to enhance peer support experiences.

### Synthesis of Multiple Signals to Identify Mental Health Problems

We found that peers identified others at risk on SNSs by synthesizing a variety of signals ranging from explicit disclosures to implicit signals. Our findings confirmed that people identify vulnerable individuals through online content and activities pertaining to interaction patterns [39], social capital [15], emotions [40], and linguistic style [41]. Research on sensitive disclosures has also characterized corpora of text [20] or imagery [2] signaling mental health problems, such as depression [40] or eating disorders [42]. In this study, we identified additional signals, such as inconsistency between online and offline personalities, which are often subtle and are detected only by those who know the poster and the context. Rather than relying on a single signal, our participants synthesized multiple signals via long-term offline and online observation to interpret a peer’s state of mind. In many cases, the individuals at risk did not explicitly solicit help or disclose problems [43]. However, their friends were able to notice the individual’s vulnerability based on implicit signals. Recognition of the signals motivated some friends to deliberately intervene in problematic situations in a timely manner.

### Attitudinal Social Distance for Identity Management

According to our survey, 66.4% (150/226) of college students said they recognized students who seemed to have mental health problems. However, a significant percentage of the participants remained inactive due to low expectations, negative beliefs about self-disclosure on SNSs, lack of confidence and knowledge, and a desire to maintain social distance from a person displaying mental health problems. While some groups of people were likely to take immediate action to persuade their suffering friends to seek help from experts, others felt fatigue and even unfollowed the poster to keep a social distance from the person displaying mental health problems. These findings align with prior work on stigma that revealed that the public desires to maintain a social distance from individuals with mental health problems [11]. Members of the public are aware of the need to help and to have prosocial reactions; however, they also consider a person with a mental health problem to be unpredictable and dangerous [11,44]. Therefore, the tendency to distance oneself from others with mental health problems was reported in Angermeyer and Dietrich [44]. Although a previous study showed that the main reason for maintaining a distance was due to induced fear (ie, fear that the person will do something violent) [11], our study findings showed that students wanted to maintain a social distance to protect their identities from stigmatization and to avoid emotional contagion. We could assume that people might feel less concerned about this fear in the online SNS context, where they can easily avoid contact from someone via lurking or disconnecting, compared with the offline environment [45], where they cannot predict where and when they will encounter a person with a mental health problem that could make him or her unpredictable and violent [11]. However, the SNS context, where interaction with someone is exposed to everyone [46], makes one more sensitive to one’s social distance from the problematic individual viewed by others. Therefore, interacting with the stigmatized content in a place where every interaction is exposed to others is considered to be careless behavior [47]. Consistent with Link et al [11] and Phelan et al [48], our study participants wanted to maintain a social distance from the content or the person who posted stigmatized issues, to protect their identities [49] and present a “positive” image on SNSs [50].

### Design Implications for SNSs to Serve as Platforms That Facilitate Support Exchanges Among Peers

#### Overview

Drawing on the results of this study about students’ experiences, perceptions, and attitudes toward peers signaling mental health problems on SNSs, we discuss opportunities for using design to enhance peer support experiences. In this section, we propose the design implications of strengthening the positive aspects of SNS usage for facilitating support and overcoming the challenges and risks that were identified and presented in the Results section.

#### Designing Peer-Supported Risk Identification

Research has suggested that computational algorithms can model behaviors of the mental health–challenged population using a variety of signals on SNSs [15,23,39,40,51]. Our study of viewers’ perspectives could complement the computational applications by incorporating implicit signals that only another person might recognize and by synthesizing these signals to provide appropriate support specific to the individual involved. We acknowledge that the majority of students might not have received training and have no qualification to diagnose others. Nevertheless, identification is the first step to access both personal support and professional treatment [52]. In addition to recognizing friends at risk, our participants responded to their peers’ signals based on context and personality. One participant’s persuasive intervention drove her close friend, who was displaying depressive symptoms, to a clinical service. We suggest that students’ contextualized sensemaking abilities could provide insight to advance computational models so they can recognize individuals who appear to be experiencing mental health problems. Furthermore, the students’ special capabilities, such as persuasive powers, could help their peers at risk become aware of their mental health conditions and could reduce barriers to help-seeking.

#### Make the Visibility of Supportive Interactions Adjustable

SNSs make activities visible to many other people [46]. Therefore, the line between public and private communication is blurring [53]. In this study, this broadcasting nature of SNSs makes every interaction transparent and, thus, it causes a reason people to hesitate to interact with or support people who are considered to have mental health problems. Both survey and interview participants reported that they felt uncomfortable responding to posts that displayed mental health problems because of the public nature of SNSs that might negatively affect their identity. For example, an audience group who can see supporters’ comments are dependent on the publisher of the original post. In other words, the supporter cannot predict the scope of the people who can see the comments [46]. This means that people sometimes bear the cost of identity management risk in the process of providing supportive comments on posts. Thus, supportive methods of interaction on SNSs need to be designed to control the likelihood of broadcasting. One can argue that fostering the use of direct communication through private messages or phone calls for the support of individuals signaling mental health problems can be considered as a primary method. However, our survey and interview study results showed that participants felt reluctant to initiate communication with someone displaying a mental health issue because they lacked confidence in their abilities to provide the appropriate support. Instead, we could consider augmenting existing features of SNSs, such as Facebook or Instagram, to be used as supportive platforms. For example, we suggest an audience management feature that enables the person who writes comments to select the audience of these comments. In existing SNS platforms, only the poster can manage this.

#### Fostering Just-in-Time Adaptive Support

The timeline and newsfeed are the main features of recent SNSs, such as Facebook, Twitter, and Instagram [54]. These features visualize a person’s activity continuously and chronologically [55]. Thus, our study participants often used these features to examine a poster’s posting pattern, which allowed them to detect problems in the poster. According to major scales of depression, such as the Center for Epidemiologic Studies Depression Scale, the frequency of depressive symptoms or negative thoughts is a basis for diagnosis [56]. The timeline and newsfeed visualizing the recent activities and thoughts of a poster experiencing mental health issues could help both posters and viewers to be aware of the poster’s situation. For those who are willing to support others, this feature also allows them to recognize their friends’ statuses and provide just-in-time support. We could consider a computerized risk notification system for those participants who are willing to support others. This system could provide a nudge to a supporter when his or her peers are uploading signals of vulnerability. These features can help to reduce the risk that the supporter will miss out on a friend’s dangerous situation. However, monitoring the sensitive SNS posts of others can be ethically problematic, especially if this is done to detect vulnerable states. Therefore, ethical consideration is needed, such as an approval system requiring mutual consent, to activate such a feature. Facebook now offers a feature that reports on users who express behavior that suggests suicide or self-harm [27], which could save a friend in an emergency. However, such a system has limitations when it comes to supporting peers, so recognizing and providing appropriate help for the intermittent symptoms of mental illness, such as intermittent depressive mood and feelings of frustration and despair, is critical.

### Limitations and Future Work

In this study, we were not able to determine whether the posters that our participants mentioned had actually been experiencing mental health issues or whether the reported activities were actual signals of mental illness. Our study did not focus on finding general factors to recognize or predict mental health conditions. Rather, our attention was on characterizing references to mental health concerns regardless of the posters’ diagnostic states. We reported empirical evidence regarding the viewers’ perceptions of the signals from the posters’ disclosures. By acquiring ground-truth data, future studies should address what factors affect the possibility of a viewer’s correct detection or false alarm. While our main study target was a student population, we recruited students from different universities to achieve diversity in the participant group. However, we conducted this study in a single country; thus, our study’s generalizability could be limited. We acknowledge that cultural background may affect how participants perceive mental health or social support. Nevertheless, the level of awareness, stigma, and literacy that the general public has about mental health issues varies significantly from country to country, which makes it challenging to derive guidelines that can be generalized to all cultural backgrounds. Therefore, it would be worthwhile for future research to compare students’ behaviors and thoughts regarding our research topic in various cultures.

### Conclusions

The goal of this study was to examine students’ perceptions of mental health–related issues displayed on SNSs. The characterization of SNS posts and activities related to negative, sensitive disclosures illuminated the key signals that students perceived as mental health problems. Our student participants synthesized multiple signals gained both offline and online to recognize an individual’s mental health condition and, further, to help them in a timely and appropriate manner. However, our study also revealed that a considerably large number of students did not provide support due to practical or emotional challenges resulting from low expectations, negative belief about self-disclosure, lack of confidence and knowledge, and a desire to maintain a social distance. Based on our findings, we provided design implications of strengthening the positive aspects of SNS usage for facilitating support and overcoming the challenges.

## Appendix

Multimedia Appendix 1 Survey questions and possible responses.

Multimedia Appendix 2 Interview guide.

## Abbreviations

IITP: Institute for Information & Communications Technology Planning & Evaluation
MSIT: Ministry of Science and ICT
SNS: social networking site
