# Supplementary figures and images for: Understanding University Students’ Experiences, Perceptions, and Attitudes Toward Peers Displaying Mental Health–Related Problems on Social Networking Sites: Online Survey and Interview Study
Source: JMIR Ment Health. 2021 Oct 5;8(10):e23465. doi: 10.2196/23465 (PMC8527375; doi:10.2196/23465)

**Survey Questions and Answer type**


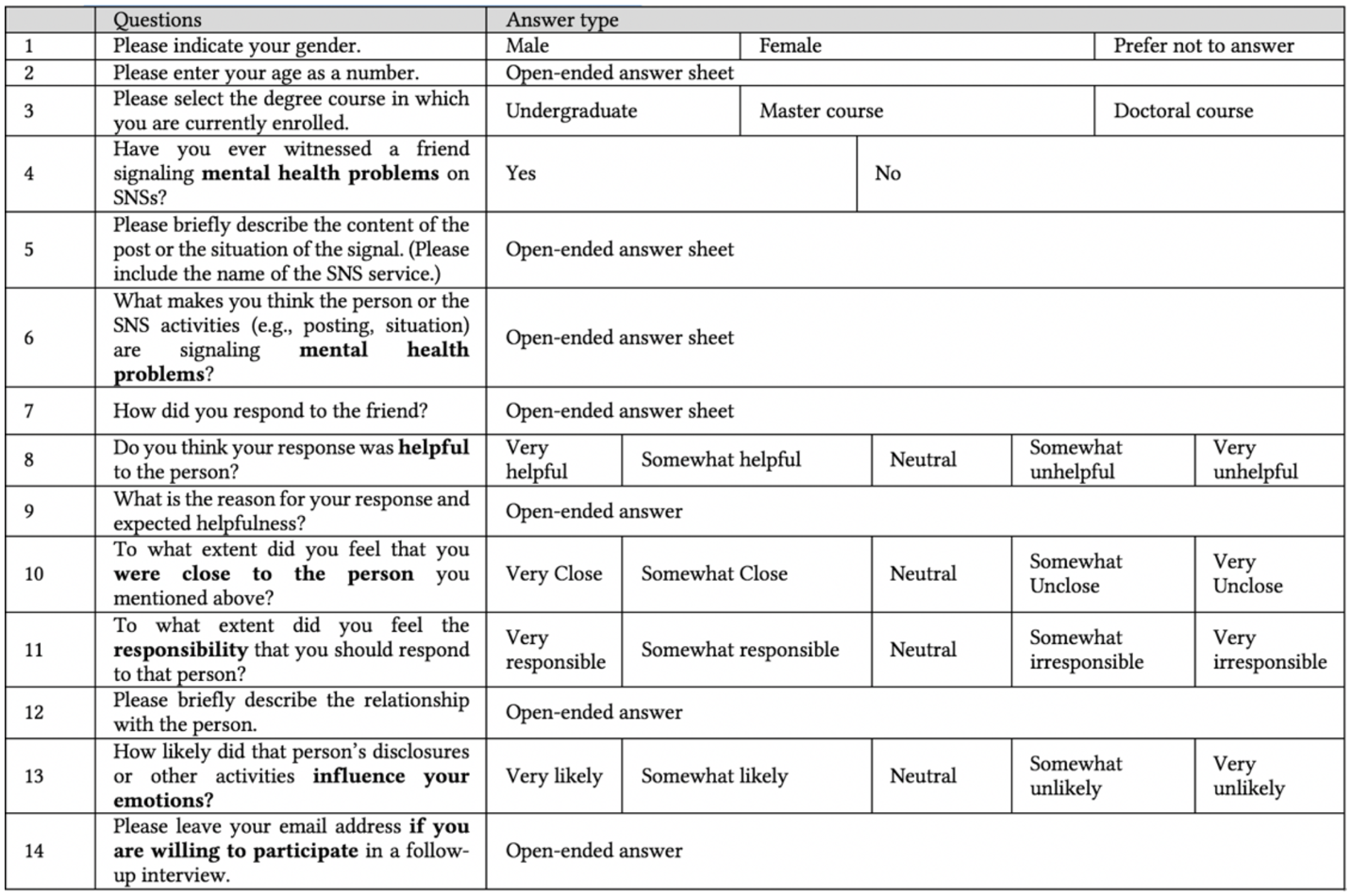

Supplement: Multimedia Appendix 1 [file mental_v8i10e23465_app1.docx]
