# Supplementary material for: Understanding University Students’ Experiences, Perceptions, and Attitudes Toward Peers Displaying Mental Health–Related Problems on Social Networking Sites: Online Survey and Interview Study
Source: JMIR Ment Health. 2021 Oct 5;8(10):e23465. doi: 10.2196/23465 (PMC8527375; doi:10.2196/23465)
Supplement: Multimedia Appendix 2 [file mental_v8i10e23465_app2.docx]

**Interview Guide**

| **Question domain** | **Questions** |
| --- | --- |
| Introductory questions | What kind of social media are you using?  What is the primary purpose of using SNS?  Which mental health disorders and problems do you know about? |
| Describing the post | Please describe the posts mentioned in the survey in detail. (If participants find it difficult to remember, give them time to browse the posts)  Was the post limited to a single occasion? Were similar posts uploaded multiple times? |
| Relationship with the poster | What is your relationship with the poster?  What do you know about the mental health problems the poster is experiencing?  When you saw the post, what did you think or feel about the author?  How does your relationship with the poster affect your reaction? |
| Reaction and rationale | Why do you think he/she uploaded the post?  Please explain in detail how you started to become concerned about the poster's mental health in relation to the post.  How did you react to the post and why?  Are there any factors that made responding to the post difficult?  How do you think your support made the poster feel?  Have you ever received such support?  Did your experience and knowledge affect your reaction and support? |
| Influence on and feelings of the participants | When you saw the post, what impact did it have on you?  How did you feel while seeing the post?  Have you ever sympathized with the content of the post or the poster? (If you have, how did it affect your reaction?) |
| Open-ended questions | If you have any good ideas or can suggest features that you would like to have to help a friend who is struggling with mental health problems on social media, please express them freely. |
